# LIBS assisted PCA analysis of multiple rare-earth elements (La, Ce, Nd, Sm, and Yb) in phosphorite deposits

**DOI:** 10.1016/j.heliyon.2023.e13957

**Published:** 2023-02-23

**Authors:** Amir Fayyaz, Haroon Asghar, A.M. Alshehri, Tahani A. Alrebdi

**Affiliations:** a National Centre for Physics, Quaid-i-Azam University Campus, 45320 Islamabad, Pakistan; b Department of Physics, King Khalid University, P.O. Box 9004, Abha 61413, Saudi Arabia; c Department of Physics, College of Science, Princess Nourah Bint Abdulrahman University, P.O. Box 84428, Riyadh 11671, Saudi Arabia

**Keywords:** Phosphorite deposits, CF- LIBS, REEs, PCA, And EDX

## Abstract

In the present study, the immediate detection of rare-earth elements (REEs) in phosphorite deposits has been reported using laser-induced breakdown spectroscopy (LIBS). Numerous emission lines corresponding to the REEs such as lanthanum (La), cerium (Ce), neodymium (Nd), samarium (Sm), and ytterbium (Yb), have been detected in the emission spectra of phosphorite-induced plasma plume. For the quantitative analysis, we employed the calibration-free LIBS (CF-LIBS), and Energy Dispersive X-ray (EDX) spectroscopy techniques. The results obtained using the CF-LIBS technique show excellent agreement with that obtained by EDX. Besides principal component analysis (PCA) was employed by incorporating the LIBS spectral data of rare earth phosphorite rocks samples containing La, Ce, Nd, Sm, and Yb emission lines. The first three PCs were observed using LIBS spectral data set showing a covariance (interpretation rate) up to 76.3%. This study suggests that LIBS yields a quick and very reliable qualitative and quantitative analysis of REEs in any geological ore sample.

## 1 Introduction

The international union of pure and applied chemistry (IUPAC) has classified rare earth elements (REEs) into a set of 17 components that are chemically analogous to those appearing in the periodic table such as lanthanides from ^57^La to ^71^Lu including scandium (^21^Sc) and yttrium (^39^Y). Their numerous properties such as luminescent (Eu, Y, Er, Nd), electrical (La, Ce, Nd, Pr), catalytic (La, Ce), and magnetic (Nd, Dy, Sm) makes them discovered simultaneously in geological deposits typically in oxide compounds. Due to the diverse applications and market dynamic contrast of rare earth metals, they are often broken up into two groups as light and heavy rare earth elements (L-REEs & H-REEs), according to their atomic weight. Interestingly, scandium is usually fallen beyond this classification and found separately from other REEs due to its different physical and chemical properties [1]. However, according to IUPAC classification, the elements starting from lanthanum (La) to europium (Eu) are the L-REEs, and the elements starting from gadolinium (Gd) to lutetium (Lu) and yttrium (Y) are the H-REEs. In European counties, the L-REEs are classified as La to Sm, and the H-REEs are categorized as Eu to Lu along with Y, whereas in China, samarium (Sm) is considered an H-RE. Hence, there is no solid limitation in the description of L-REEs and H-REEs [2]. In the latest years, the strategic, economic, and engineering significance of REEs such as La to Lu along with Sc, and Y has increased owing to the application in a large range of fabrication of goods in industries. REEs have high mechanical strength and high melting point, so they are widely used in modern electronics, high-power fiber lasers, batteries, magnets, catalysts, hybrid vehicles, mobile phones and laptops, miniature incandescent light tubes, radar systems, and rechargeable batteries [3,4,5].

Various analytical techniques such as X-ray fluorescence (XRF), inductively coupled plasma mass spectrometer (ICP-MS), inductively coupled plasma atomic emission spectrometer (ICP-AES), atomic-absorption-spectrometer (AAS), and instrumental-neutron-activation analysis (INAA), have been utilized for qualitative and quantitative analysis. Besides, ICP-MS and ICP-AES have extensively implemented techniques due to high-grade precise, and accurate qualitative and quantitative analysis of the target sample [6]. However, these techniques take too much time for the sample preparation procedure and have a problem of spectral interference effect due to the presence of multi-elements in the target sample [7,8]. The problem of the spectral interference effect can be reduced using high-performance liquid chromatography in the analysis but additional expensive instruments are needed along with a long time-consuming sample preparation [9].

Laser-induced breakdown spectroscopy (LIBS) is a developing spectroscopical analytical tool for the qualitative and quantitative study of various samples such as cement [10,11], steel [12], soil [13,14,15], pharmaceutical tablets [16], plants [17], aqueous solution [18,19], seaweed fertilizer [20] and neoplastic tissues [21]. It is a fast and globally responsive technique that requires minimal sample pre-treatment as required in conventional techniques such as ICP-MS and ICP-AES. In LIBS, calibration-free (CF) and calibration curve (CC) techniques are commonly employed for the quantitative analysis of the sample under investigation. In the CC-LIBS technique, the reference or standard target samples are used and calibration curves are constructed among the line intensity and the known chemical concentration of the standard or reference samples. The unknown sample concentration can be determined by fitting line intensity on the standard calibration curves [22,23]. The drawback of this technique is that the target samples with a concentration analogous to the unknown samples are essential which is difficult to achieve in each case. To avoid this problem, the CF-LIBS technique has been demonstrated [24,25]. Stoichiometric ablation, optically-thin plasma, and local-thermodynamical-equilibrium (LTE) conditions are essentially required for the CF-LIBS quantitative analysis [26,27].

Previously reported studies on rare earth elements generally concentrated on standard samples or compounds with pure oxides. Numerous research articles have explained the study of rare earth elements in different conditions by applying the LIBS technique. Unnikrishnan et al. [30] performed the quantitative analysis of La and Nd in metal oxysulphide phosphors with five different chosen concentrations of Nd in (La_1-x_Nd_Y_)_2_O_2_S and La samples using a calibration-based method. The linear-fit (R^2^) regression coefficients for La and Nd were achieved as 0.99, and 0.98, respectively. Alamelu et al. [31] determined the REEs including Eu, Gd, and Sm present at trace levels with an accuracy of 5% using the LIBS technique. Furthermore, Dwivedi et al. [32] have studied quantitative analysis along with the plasma parameters of REEs (Nd, Er, Eu, Ho) in doped oxy fluoroborate optical glasses using the plasma spectroscopy technique. Moreover, the capability of the LIBS method has been reported to evaluate the heavy elements such as Eu, Ba, Ho, Ca, Nd, and Er as well as light elements such as O, F, and B that exist in the oxy fluoroborate optical glasses. Devangad et al. [33] have determined the elemental composition of REEs (Sm, Tm, Yb) in doped-phosphate-glasses at four various concentrations of 10%, 5%, 2%, and 1% by applying a LIBS technique. The authors have assessed the LIBS analytical studies through the regression-coefficients near unity (0.99). Phuoc et al. [34] have analyzed REEs present in the ash-samples from Powder-River-Basin sub-bituminous coal (PRB-coal) using LIBS and elements were identified in the lanthanide series (Ce, Eu, Ho, La, Lu, Pr, Pm, Sm, Tb, Yb) and in the actinide series (Ac, Th, U, Pu, Bk, Cf). Castro et al. [35] have investigated the direct computation of base (Fe and B) and some REEs (Tb, Sm, Pr, Nd, Gd, and Dy) in hard-disk magnets using five calibration strategies. Bhatt et al. [36] have reported the multivariate and univariate analyses of six REEs (Y, Sm, Nd, Gd, Eu, Nd, and Ce) using the LIBS technique while the samples of binary mixtures were made utilizing alumina (Al_2_O_3_) matrix with contrasting concentrations of the oxides of REEs. Abedin et al. [37] analyzed the raw monazite sands, collected from the beaches of Bangladesh using the LIBS technique and detected some rare earth lanthanides) such as Er, Dy, Gd, Yb, Y, Nd, Pr, La, and Ce including other metallic ingredient elements such as Zr, Cr, Ti, Mg, Mn, Nb, and Al. Manard et al. [38] have used a handheld LIBS instrument for the qualitative analysis of REEs (Nd, Eu, and Yb) in a uranium oxide matrix (U_3_O_8_). Most recently, Gaft et al. [39] detected REEs using diatomic molecular laser-induced plasma spectroscopy. However, the above-mentioned circumstances show that significant work has been presented on the qualitative analysis of REEs and quantitative analysis of the standard samples. Hence a compositional analysis of the REEs plays a significant role in describing rare earth raw samples. By considering the above-mentioned analysis, the current study establishes the significance of the LIBS tool coupled with PCA to perform qualitative, quantitative, and classification analyses of rare earth phosphorite rocks samples. Further, this work emphasizes future REEs research to distinguish variations in the natural rare earth resources which are highly related to the geological and ores samples study and the economic power of a country.

The main motivation behind the present work was to achieve the chemical and classification analysis of rare-earth-based phosphorite rocks. To the best of our knowledge, this is the first time a comprehensive analysis of the phosphorite rock (PR) samples using LIBS coupled with chemometric analysis. In this work, 5 different phosphorite rock (PR) samples were investigated using the LIBS analytical technique. The quantitative as well as qualitative measurements of the REEs identified in the PR samples were performed using the LIBS analysis. For the qualitative investigations, the LIBS spectra of the samples were detected using the NIST database [40]. The quantitative analysis of the samples under investigation was achieved through CF‒LIBS, supplemented by the EDX analysis showing an excellent agreement. To detect rare earth patterns, we have coupled LIBS spectral data to PCA analysis to examine the REE distribution in the samples that might be associated with mineralization. For the clustering, and classification, PCA analysis was carried out using LIBS spectral data of the PR samples based on the optical emission lines of La II 489.991 nm, Ce II 422.260 nm, Nd I 468.345 nm, Sm II 392.240 nm, and Yb II 328.937 nm for S1, S2, S3, S4, and S5 samples, respectively. The first three PCs described 76.3% of the variation with PC1 at 40.70%, PC2 at 22.40%, and PC3 at 13.20%. This work demonstrates the potential of LIBS in combination with EDX, and PCA to characterize and quantify the rare earth samples and also provide assistance to the geologists.

## 2 Materials and methods

**Fig. 1:**
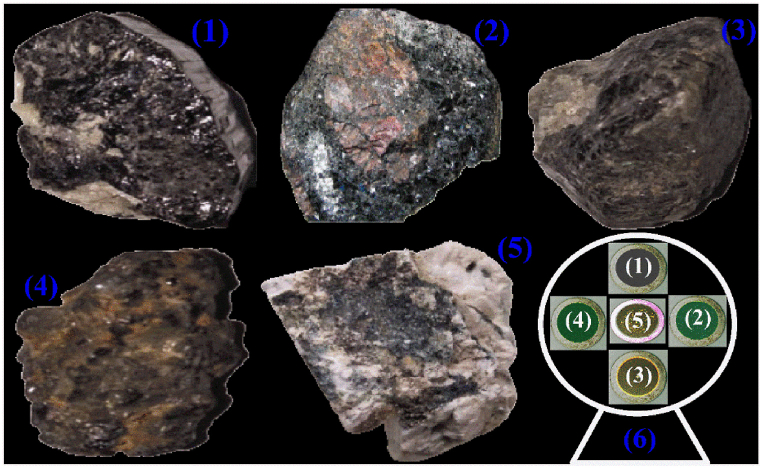
Phosphorite rocks samples (1–5) and (6) show the pallets of the corresponding samples containing fine ground powder.

A total of five PR samples were collected and were formerly assessed by a professional local geologist. Fig. 1 shows the photographs of the investigated phosphorite rocks. The phosphorite samples were grinded initially by a ball-crushing system. To make a fine powder, samples were further grinded manually using a mortar pestle. The mortar pestle was first washed with purified water, dried in an electric drying oven at 80 °C to eliminate moisture contents, and then cleaned with ethanol to prevent any impurities. Polyvinyl alcohol (PVA) having chemical molecular formula (C_2_H_4_O)_x_ was used in powder form as a binder to improve cohesion. For each sample, 5 g of sample powder was mixed with 0.4 g of PVA with 4 ml of boiling filtered water (H_2_O). The composite material was poured into a synthetic die after being stirred and left dry at room temperature till it was hardened. Finally, pallets of the samples having 1.5 cm diameter and 4 mm thickness were made employing a pellet presser at 50 tons for 20–30 min.

## 3 LIBS experimental arrangement and detection parameters

**Fig. 2:**
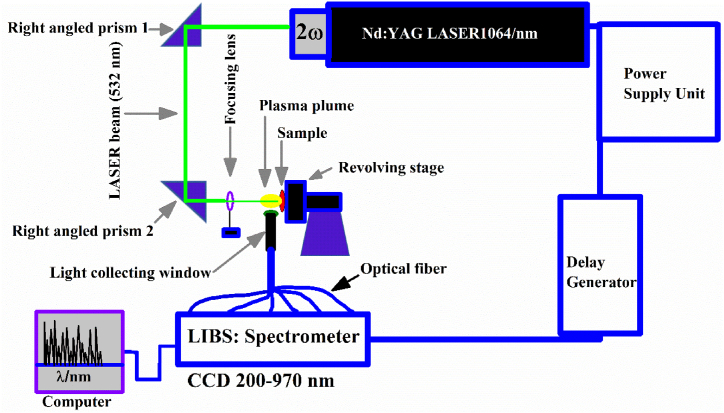
LIBS experimental setup for the optical emission study of phosphorite rocks.

A graphic illustration of the LIBS arrangements is presented in Fig. 2. A detail about the experimental system is explained in our previously reported articles [41,42]. A Q-switched Nd: YAG, 2nd harmonic (2ω) high-power Laser (Brilliant-B Quantel, France) having 400 mJ energy at 532 nm, comprising 5 ns pulse-width, and 10 Hz frequency was employed to generate a micro-plasma on the target surface. The laser pulse energy was adjusted by changing the Q-switch delay and was measured using the NOVA-QTL energy meter. The time-integrated LIBS experimental arrangement was accomplished at ∼100 mJ laser energy with a 10 μs integration time and a 2 μs delay time between the acquisition system and the laser pulse. A quartz convex lens having a focal length of 20 cm was employed to collimate the laser-beam on the sample surface. The diffraction-limited diameter of the laser beam was calculated using Eq. (1) [43].

(1) $$d=2.44\times \left(\frac{\lambda \times f}{D}\right)$$

Where d represents the beam diameter at the focus of the lens, *λ* indicates the optical wavelength of the laser, f denotes the focal length of the lens and D implies the diameter of the laser beam before focusing. The observed spot diameter was ∼0.05cm which corresponds to a laser fluence of about 51 Jcm^−2^. To avoid inhomogeneity, the PR samples were positioned on a revolving stage and registered the emission spectra from different spots on the fresh surface of the target. The sample was kept at a distance, less than the focal length of the lens to avoid air breakdown near the surface of the sample. The plasma emission light was collected through a collimating lens (0°–45° field of view) at a spectrometer (Avantes, Holland) which is coupled to an optical fiber having high–OH, and a core diameter ≻of 550 μm. The spectrometer consists of 6 miniature channels each having a 10 μm slit width, comprising the wavelength range from 200 nm to 970 nm. The instrumental resolution of our system (0.06 ± 0.01) nm at 600 nm was established from the full width at half maximum (FWHM) of sharp emission lines of neon emitted by a low-pressure neon-filled hollow cathode lamp. The AvaSoft 8.5 was employed to analyze the optical spectrum. This software allows the detection of optical spectra, the rectification of background signals, and the area integration of the emission lines. To register the LIBS emission spectrum, a background spectrum of the six miniature spectrometers was recorded and then laser energy was optimized to get a high signal-to-noise ratio of the emission spectrum. Subsequently, the optical spectra of all the samples were then recorded as an average of 20 laser shots at various points on the sample surface. The background spectrum was then deducted from the registered spectrum to get a noise-free emission spectrum.

## 4 Results and discussion

### 4.1 Emission studies of rare earths in phosphorite rocks subject to LIBS

**Fig. 3:**
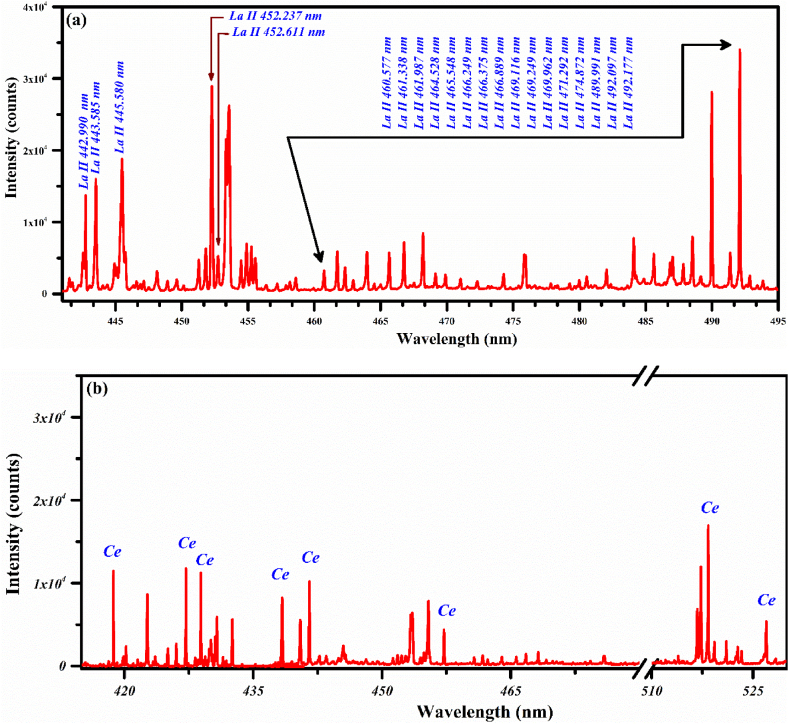
LIBS spectra of the PR samples: (a) shows a spectrum of the PR sample S1 containing rich emission lines of La, and (b) shows a spectrum of PR sample S2 holding various emission lines of Ce.

**Fig. 4:**
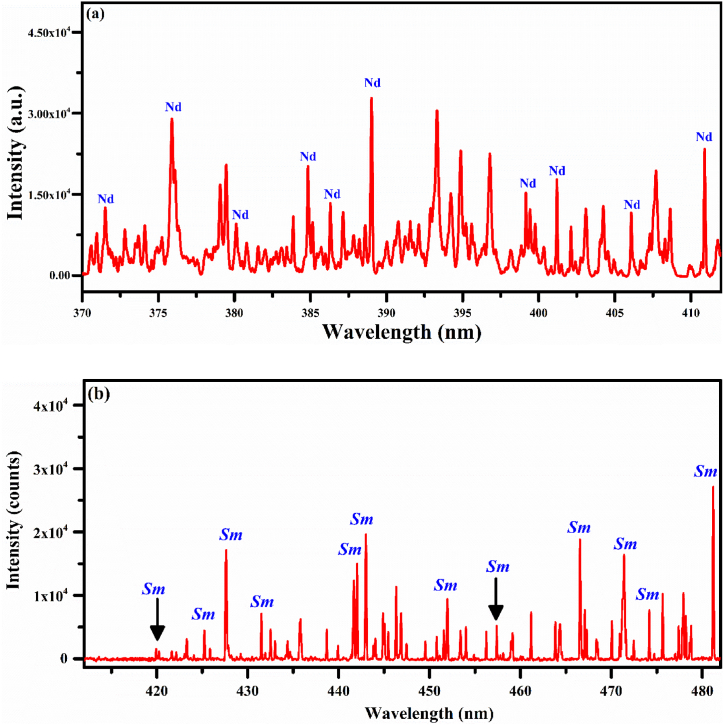
(a) Shows a spectrum of the PR sample S3 along with emission spectral lines of Nd, (b) shows a spectrum of the PR sample S4 comprising rich spectral lines of Sm.

LIBS optical emission spectra of rocks having REE traces are remarkably dense due to the rich emission lines of REEs and are identified using the NIST database [40]. The optical emission spectra of the PR samples (S1 to S5) covering the wavelength region from 200 nm to 970 nm are demonstrated in Fig. 3, Fig. 4. The characteristic emission lines of the rare earth metals are detected in samples S1 to S5. Owing to the spectral interferences, only dominating lines belonging to different REEs including La, Ce, Nd, Sm, and Yb are labeled. The measurements of the spectral lines yield qualitative and quantitative information regarding the occurrence of trace and major elements. The identification of the spectral lines was performed with the help of the NIST-Database [40]. Since the plasma was produced in the air environment, the emission lines of oxygen, hydrogen (H_α_), and nitrogen are also detected in the optical emission spectra. To elaborate on the persistent spectral lines of Yb, Sm, Nd, Ce, and La in the spectra of rare earth samples, we present different sections of the optical emission spectrum in Fig. 3, Fig. 4 under identical LIBS parameters. In addition, we select only those spectral regions where spectral interferences of the elements are minimal.

In Fig. 3 (a), we present the optical emission spectrum of the PR sample (S1) covering the wavelength region from 440 nm to 495 nm. The spectrum of the sample (S1) displays characteristic spectral emission lines due to their numerous elemental compositions. However, it is important to highlight the spectral transitions of the REEs in the sample. Only the dominating or strong emission lines belonging to La are highlighted in this section and labeled according to their configuration. The resonance line profiles of the singly ionized lanthanum at 443.585 nm (4f5d ^3^D_3_ → 5 d^2^ ^3^F_2_), 466.249 nm (4f5d ^3^D_1_ → 5 d^2^ ^3^F_2_), and 489.991 nm (4f5d ^3^G_3_ → 5 d^2^ ^3^F_2_) confirm the presence of La in the PR sample (S1). The classifications of the spectral emission lines yield qualitative as well as quantitative information about the occurrence of major as well as trace elements. Therefore, the characteristic emission lines of La are observed as dominant followed by Ti, Ca, Fe, Si, Al, Mg, and Na in the sample (S1). In the PR sample (S2), several optical emission lines of Ce are detected from 415 nm to 530 nm as shown in Fig. 3(b). In this region, the main emission lines of Ce I at 418.73, 422.77, 427.02, 428.87, 438.22, 441.87, 457.23, 518.75, 521.19, and 526.57 nm have been identified showing the presence of cerium in this sample.

In Fig. 4(a), we describe the optical emission spectrum of the PR sample (S3) covering the spectral range of 370 nm–415 nm. Several spectral lines are detected in this spectrum, particularly around 385 nm, 389 nm, 401 nm, and 411 nm, and marked in blue color which qualitatively establishes the existence of Nd in the target sample. The major emission lines of Nd I have been identified at 463.42, 488.38, 489.69, 492.45, 494.48, and 495.48 nm. It is however worthwhile to mention that the strong emission lines of Ti I appears at 376.34 nm due to the 3 d^3^(^4^F)4p ^5^G_3_ → 3d^2^4s^2^ ^3^F_2_ transition and Ca II at 393.37 nm belonging to the 3p^6^4p ^2^P_3/2_ → 3P^6^4S ^2^S_1/2_ transition. In Fig. 4(b), we show the sample (S4) spectra covering the optical wavelength region from 412 nm to 482 nm. In this region, the main spectral lines of singly-ionized samarium (Sm II) at 420.30, 421.03, 423.67, 425.64, 427.96, 428.07, 429.67, 431.89, 442.05, 442.11, 442.43, 443.38, 443.43, 451.96, 457.76, 466.93, 466.96, 470.44, 471.98, 474.57, 481.58 nm have been identified demonstrating the presence of Sm in a sample (S4).

**Fig. 5:**
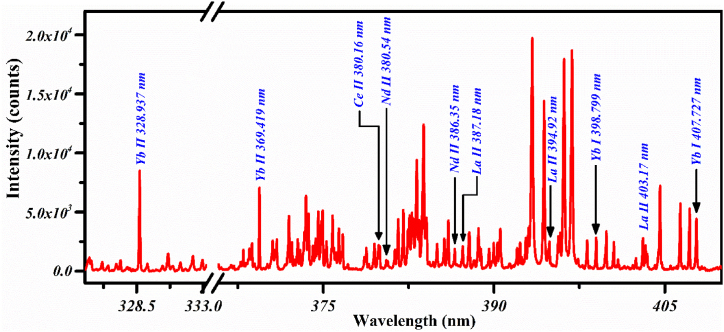
The emission spectrum of the PR sample S5 including optical emission lines of Yb, Ce, Nd, La, Al, and Ca.

In Fig. 5, we present the spectrum of the PR sample (S5) covering the wavelength ranges from 325 nm to 410 nm. A strong emission line at 328.937 nm is identified in the wavelength section from 325 nm to 333 nm corresponding to singly ionized Yb (II) due to the transition 6p ^2^P_3/2_ → 6s ^2^S_1/2_. In sections 365-410 nm, various emission lines are observed which correspond to REEs such as Yb, Ce, Nd, and La along with Al & Ca. The resonance line profiles of the singly-ionized lines of Yb (II) (at 369.419 & 398.799 nm) are again revealing their presence in this region. The spectral lines of Yb II at 369.419 nm and 398.799 nm occur due to the transitions; 6p ^2^P_1/2_ → 6s ^2^S_1/2_ & 6s6p ^1^P_1_ → 6s^2^ ^1^S_0_ respectively. The presence of cerium (II) spectral emission line profile at 380.160 nm due to transition 6p ^4^H_13/2_ → 6s ^4^G_11/2_ shows the cerium concentration in the sample (S5). Likewise, other REEs such as La (II) at; 387.18 nm, 394.92 nm, & 403.17 nm and Nd (II) at; 380.54 nm, & 386.35 nm demonstrate their presence in the sample (S5).

### 4.2 Plasma characterization

The electron number density was calculated using a Lorentzian fitted line profile full-width at half-maximum (Δλ_FWHM_) of the hydrogen H_α_ line at wavelength 656.28 nm using Eq. (2). The FWHM of the line profile was calculated using a Lorentz fitting which incorporates the instrumental width (0.06 ± 0.01 nm). The recorded H_α_ emission lines of all the target samples were well-isolated having a good quality SNR letting a precise calculation of the electron plasma number-density. In this work, the hydrogen emission lines appeared owing to the PVA and air. Consequently, a self-absorption correction of the H_α_-line was made. As self-absorption generates a broadening in the Stark-width of H_α_ spectral line which makes an error in the estimated value of the electron-density. The observed average Lorentzian FWHM of the H_α_ spectral lines of all the 5 PR samples was calculated as Δλ_FWHM_ = (1.3 ± 0.5) nm. The estimated Doppler width (ω_D_) utilizing excitation temperature was ∼0.020 nm and is negligible compared to Lorentzian width (ω_L_) [44,45].

(2) $$N_{e}={\left(\frac{{\Delta \lambda }_{FWHM}}{1.098}\right)}^{1.47135}\times 10^{17}{cm}^{-3}$$

**Fig. 6:**
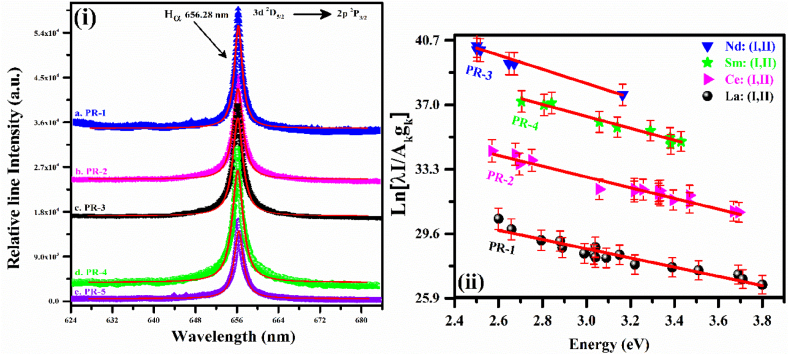
(i) The experimental line profiles for the H_α_ emission lines at 656.28 nm of the 5 PR samples along with Lorentzian fit (red solid line) (ii) Saha-Boltzmann-plots of the PR-samples (S1, S2, S3, and S4), constructed with La, Ce, Sm, and Nd atomic and ionic (I, II) lines. (For interpretation of the references to color in this figure legend, the reader is referred to the Web version of this article.)

Next, the self-absorption coefficient ($SA\approx e^{-\nu }/\nu$, ‘ν’ correspond line center) [46,47] was calculated. For an optically thin line, the self-absorption coefficient (SA) was taken as zero (SA = 0) and it grows up to 1 (SA = 1) for the completely self-absorbed line. The self-absorption effect becomes significant as ν→1 which yields SA ≥ 0.36. In the present work, the coefficients of SA were calculated to ≤0.018 for the hydrogen H_α_ emission lines for all the samples as shown in Fig. 6(i). Therefore, the self-absorption of the H_α_-lines was sufficiently low to consider, and hence it was ignored. The calculated Lorentzian FWHM of the Stark widths (ω_stark_) was in the range of 1.21–1.4 nm showing good agreement with the reported values [48,49]. The average electron-density was N_e_ = (1.31 ± 0.1) × 10^17^ cm^−3^ and was calculated by using Eq. (2). The errors were estimated from the standard deviation of the acquired values from the various target samples. All the calculated plasma number densities from the various samples were found in good agreement with the measured uncertainty. To describe the laser-induced-plasma, certain conditions including optically thin plasma and LTE must be fulfilled. McWhirter proposed a criterion to validate LTE condition in plasma which is established on the presence of a critical-electron number density $N_{c}^{e}$ such that electronic collisions dominate the radiative-processes [21,50].

(3) $$N_{c}^{e}\left({cm}^{-3}\right)\geq 1.6\times 10^{12}{\left(T_{e}\left(K\right)\right)}^{1/2}{\left({\Delta E}_{ki}\left(eV\right)\right)}^{3}$$

Where ΔE_ki_ and T_e_ are the excitation temperature and the energy difference between the states (*k→i*), respectively. The estimated electron density using Eq. (3) was about 1 × 10^15^ cm^−3^, which is less compared to the number-density measured from the stark broadened H_α_ line profile, and therefore, the condition of LTE was validated. For the temperature Boltzmann plots, plasma emission lines have to be optically thin. In order to verify whether the lines are optically thin or thick, the various observed pair of line intensities ratios were compared with that determined from the spectroscopic parameters. The resulting temperature obtained using the optically thin lines is only a population-average temperature. The following intensity ratio method has been adopted to verify the optically thin plasma condition [21,51,52]:

(4) $$\frac{I_{ki}}{I_{nm}}=\left(\frac{\lambda_{nm}}{\lambda_{ki}}\right)\left(\frac{A_{ki}}{A_{nm}}\right)\left(\frac{g_{k}}{g_{i}}\right)\mathrm{Exp}\;\left(-\frac{E_{k}-E_{n}}{k_{B}T}\right)$$

Where I_ki_ and I_nm_ are spectral-intensities of observed spectral-lines at *λ*_*ki*_ and *λ*_*nm*_ respectively. *A*_*ki*_ and *A*_*nm*_ are the reported transition-probabilities, $g_{k}$ and $g_{n}$ are the corresponding statistical-weights of upper-levels, k_B_ and T are the Boltzmann constant and the excitation temperature, respectively. To confirm the optically-thin plasma-condition, we have employed the experimentally measured line-intensities ratio of various spectral lines of La I, Sm I, and Nd I and comparing it with calculated intensities ratios using Eq. (4). In Table 1, we present all the spectroscopic data about calculated and observed intensity ratio for a pair of La I, Sm I, and Nd I emission spectral lines. The observed-intensity-ratios for the pairs of La I, Sm I, and Nd I spectral lines show an excellent agreement with the experimentally measured ratios which confirms the plasma is optically thin.

**Table 1:** Validation of optically thin plasma by evaluating theoretical and experimental intensity ratios.

REEs	*λ* (nm)	Relative Intensity (a.u.)	A_k_g_k_ (s^−1^)	E_k_ (eV)	Spectroscopic parameters $\frac{I_{1}}{I_{2}}$	Experimentally observed $\frac{I_{1}}{I_{2}}$
La I	389.86	29,646	$3.80\times 10^{7}$	3.17	0.652	0.65
La I	390.26	19,270	$2.48\times 10^{7}$	3.17		
Sm I	374.38	27,357	$2.46\times 10^{7}$	3.64	0.49	0.489
Sm I	375.52	13,404	$1.21\times 10^{7}$	3.63		
Nd I	372.24	12,526	$4.0\times 10^{6}$	3.54	0.444	0.439
Nd I	397.35	5511	$1.9\times 10^{6}$	3.54		

The plasma excitation temperature is an essential plasma parameter that can be measured using the Boltzmann-plot and the Saha-Boltzmann plot method. For the Boltzmann-plot approach, only the atomic emission lines are essential while using the Saha-Boltzmann plot approach, numerous spectral emission lines such as atomic and ionic lines are needed. The Saha-Boltzmann relation for the line emissivity of an ingredient element can be developed by utilizing the following steps:

The emission-integrated line intensities using the Boltzmann equation for two transitions having the transition probabilities, the transition wavelengths, the statistical weights of the upper-level, the energies of the upper-levels, the temperature (T), and Boltzmann's constant (k) such as (I_1_→ I_2_, A_1_→A_2_, *λ*_1_ & *λ*_2_, g_1_ & g_2_, E_1_ & E_2_, T, and k) respectively [53];

(5) $$\frac{I_{1}}{I_{2}}=\left(\frac{A_{1}}{A_{2}}\frac{g_{1}}{g_{2}}\frac{\lambda_{2}}{\lambda_{1}}\right)\exp^{\left[\frac{E_{2}-E_{1}}{kT}\right]}$$

Taking the natural log of Eq. (5) yields a linear correlation between the terms such as ln (Iλ/A_k_g_k_) along with the integrated intensities ratio, and the upper-level energies as well:

(6) $$\Rightarrow \ln\left(\frac{I_{1}}{g_{1}}\frac{\lambda_{1}}{A_{1}}\right)-\ln\left(\frac{I_{2}}{g_{2}}\frac{\lambda_{2}}{A_{2}}\right)=\frac{E_{2}-E_{1}}{kT}$$

This linear relationship (refer Eq. (6)) gives the electron temperature (slope ∼1/kT) by constructing the ln (Iλ/A_k_g_k_) term as a function of the upper-level energy. The error can be minimized to improve the accuracy of the temperature measurement using multiple emission spectral lines. However, for high temperatures, the line intensities ratios from several ionization stages can be associated with the plasma temperature using a relation similar to the Boltzmann equation. Because the energy spans in a separate ionization level at high temperatures are not too large to make precise temperature measurements. Higher energy spans are achievable by employing transitions from several ionization phases utilizing the Saha-Boltzmann's equation;

(7) $$\Rightarrow \frac{I_{1}}{I_{2}}=6.04\times 10^{21}\left(\frac{A_{1}}{A_{2}}\frac{g_{1}}{g_{2}}\frac{\lambda_{2}}{\lambda_{1}}\right)\frac{T^{\frac{3}{2}}}{N_{e}}\exp^{\left[\frac{E_{2}-E_{1}-E_{\infty }+{\Delta E}_{\infty }}{kT}\right]}$$

Here, E_∞_ is the ionization-energy, N_e_ is the electron-density, subscripts 1 and 2 refer to the different ionization stages, and ΔE is the ionization-energy correction owing to minor scale polarization of the optical plasma [54,55]. Eq. (7) is similar to the Boltzmann equation however includes the T^3/2^ factor and an electron number density. The T^3/2^ factor in Eq. (7) prevents the application of the Saha equation through multiple lines in the Boltzmann equation. A temperature measurement using the Saha equation can also be performed after simplification of Eq. (7). Eq. (7) can be simplified by taking the natural log;

(8) $$\Rightarrow \ln\left(\frac{I_{1}}{g_{1}}\frac{\lambda_{1}}{A_{1}}\right)-\ln\left(\frac{I_{2}}{g_{2}}\frac{\lambda_{2}}{A_{2}}\right)=\ln\left(6.04\times 10^{21}\times \frac{T^{\frac{3}{2}}}{n_{e}}\right)+\frac{\left(E_{2}-E_{1}-E_{\infty }+{\Delta E}_{\infty }\right)}{kT}$$

Now, Eq. (8) can be utilized to plot both the species such as atomic (I) and ionic (II) on the same Boltzmann-type plot. In Eq. (8), two major differences such as the upper-level energy difference, and the first term has been transformed. To draw atomic and ionic lines on the same plot, we adjust the abscissa values of the ionic energy levels $\left({E_{J}}^{z*}\right)$ only, by inserting modified ionization-energy such as ${E_{J}^{z}}^{*}=E_{J}^{z}+\sum_{k=0}^{z-1}\left(E_{\infty }^{k}-{\Delta E}_{\infty }^{k}\right)$ [56] to the ion energy levels and finally, we modify the Saha-Boltzmann relation for the emission line emissivity of an ingredient element as;

(9) $${\ln\left(\frac{I_{1}}{g_{1}}\frac{\lambda_{1}}{A_{1}}\right)}^{*}=\ln\left(\frac{I_{1}}{g_{1}}\frac{\lambda_{1}}{A_{1}}\right)-\ln\left(6.04\times 10^{21}\times \frac{T^{\frac{3}{2}}}{n_{e}}\right)$$

Now, we can make plots similar to Boltzmann plots for each species such as atomic as well as ionic using Eq. (9). The accuracy of temperature measurement can be improved using a sufficient number of the available atomic and ionic spectral lines having significant excitation energy differences in upper levels. Hence, the average plasma temperature was achieved from the Saha-Boltzmann plots of La (I II), Ce (I II), Sm (I II), and Nd (I II) emission lines. La, Ce, Sm, and Nd spectral lines were selected because these are the observed elements in the spectra of 5 PR samples having the largest number of spectral transitions with recognized spectroscopic data in the optical spectral-range from 200 to 970 nm. In Saha‒Boltzmann-plots, the data-points of La, Ce, Sm, and Nd emission lines were almost linearly distributed with R^2^ ≥ 0.97, as presented in Fig. 6(ii). The average-temperature was kT_La,Ce,Sm,Nd_ = (0.90 ± 0.02) eV. The vertical error bars (red color) in Fig. (ii) show the standard deviation in the data points.

By using the criterion given by Cristoforetti et al. [57], the LTE-condition has also been validated by approximating the diffusion-length for an inhomogeneous plasma. The diffusion-length was estimated using the following relation [21,57,58].

(10) $$\lambda \approx \sqrt{D_{diff}\times \tau_{rel}}=1.4\;x10^{12}\;\left[\frac{{\left(k_{B}T\right)}^{\frac{3}{4}}}{N_{e}}\right].\;{\left(\frac{\Delta E}{M_{A}f_{12}\;\left(\bar{G}\right)}\right)}^{\frac{1}{2}}.\exp\left[\frac{\Delta E}{2k_{B}T}\right]$$

(11) d≈T(x)(dT(x)dx)−1

Where k_B_T and ΔE in Eq. (10) are determined in electron volt (eV), the number-density N_e_ is measured in cm^−3^, M_A_ is the atomic mass of a specie, $\bar{G}$ indicates the gaunt factor, f_12_ indicates the oscillator-strength [40], d in Eq. (11) shows the plasma-diameter (few mm) and also called as characteristic variation-length [58]. In this study, the emission-line of neutral calcium (Ca I) were employed to calculate the diffusion-length. The diffusion length was calculated to be $\lambda ≅2.1\times 10^{-3}\;mm$ which is much less relative to characteristic-variation-length of plasma (10λ<d) that confirms the plasma is very close to LTE. Once plasma satisfy the conditions of LTE and optically-thin; the plasma-parameters can be used to estimate composition of elements using CF‒LIBS.

### 4.3 The effects of spatial and laser irradiance on plasma parameters

**Fig. 7:**
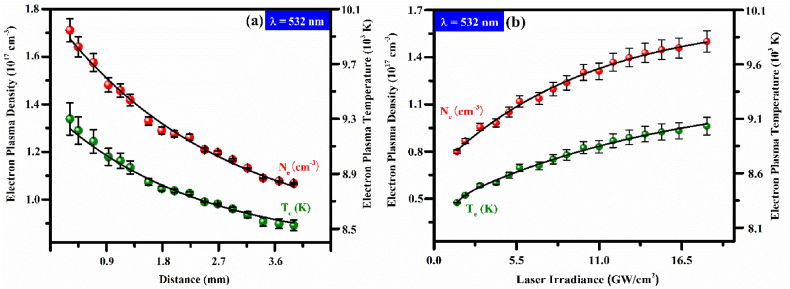
(a) Spatial distribution of electron plasma density (cm^−3^) and plasma temperature (K) of PR sample-(S1) plasma, (b) Varying trend in the electron density and electron temperature of PR sample-(S1) plasma versus laser irradiance from 0 to 22 GW/cm^2^ of 532 nm of Nd: YAG laser.

In the following section, we investigated the behavior of the electron number density and electron temperature by varying the distance of the propagation-direction of the plasma-plume from the surface of the target sample. Fig. 7(a) shows the spatial variation of plasma parameters of PR sample-(S1) plasma along the plume size up to 7.0 mm at under 12 GW/cm^2^ laser energy. At 532 nm laser wavelength, the temperature reduces from 9300 to 8500 K. A high temperature near the sample is owing to higher plasma growth and cooling rates as well, while at 3.5 mm from the surface of the target material, the recombination mechanism controls the plasma decay [59,60,61]. This kind of pattern is already described in LIBS experiments [60,61]. Likewise, the spatial distribution of the electron number density in red spheres is demonstrated in Fig. 7(a). The maximum value of electron density near the target surface is calculated as $1.7\times 10^{17}$ cm^−3^, which reduces up to 7.0 mm. The vertical error bars (black color) show the standard deviation in the data points. More energy is submerged in the sample surface because of the lowest distance prompting helpful ionization, while by expanding distance recombination starts that adjusts the ionization level and lessens the electron plasma density.

The laser-irradiance dependence of PR sample-(S1) plasma is presented in Fig. 7(b). The solid black curve is showing the power law fitted pattern in electron density and electron excitation temperature data points versus laser irradiance from 0 to 22 GWcm^−2^. At 532 nm laser wavelength, the electron temperature increases from 8300 to 9000 K. The plasma temperature rises up to 11 GW/cm^2^ and subsequently, saturation impact begins happening as a result of plasma shielding. The plasma temperature described in literature [62] at 10.6 GW/cm^2^ using a 532 nm laser agrees well with the present work. Likewise, the electron density (red spheres) versus laser irradiance from 0 to 22 GW/cm^2^ is drawn in Fig. 7(b), which shows a growing curve from $0.8\times 10^{17}$ to $1.55\times 10^{17}$ cm^−3^ at laser wavelength 532 nm. The solid black curve is the power law fitting, shows the growing pattern with laser energy. Liu et al. [63] studied significantly high plasma density and linear growth up to 55 GW/cm^2^.

### 4.4 Quantitative analysis using CF‒LIBS

We have employed the CF–LIBS method to estimate the chemical concentration (μg/g) of the 5 PR samples, which is studied in detail somewhere else [24,26,27]. The atomic concentration ($C^{\gamma }$) of the elements exist in the sample are estimated utilizing the Boltzmann equation [64,65].

(12) $$FC^{\alpha ,\gamma }=I_{ki}\frac{Z^{\alpha ,\gamma }\left(T\right)}{A_{ki}g_{k}}\exp\left[\frac{E_{k}}{k_{B}T}\right]$$

Here, $Z^{\gamma }\left(T\right)$ in Eq. (12) is the partition function that is defined as $Z^{\gamma }\left(T\right)=\sum_{i}g_{i}e^{-\frac{E_{i}}{k_{B}T}}$ [40], $C^{\gamma }$ is the concentration of the neutral atom, factor F is associated with the ablated mass and volume and constant for constant efficiency of the spectral system. The experimental factor F can be estimated by normalizing the total concentration of all the elements to unity such as $\sum C_{tot}^{\alpha ,\gamma }=1$ [21,65], $g_{k}$ correspond to statistical weight [40], $I_{ki}$ is the integrated transition spectral line intensity, $A_{ki}(s^{-1}$), E_k_ (eV), T, E_k_ (eV), and k_B_ is the reported transition probability, the excitation temperature, the upper-level energy, and the Boltzmann constant, respectively. In order to reduce the relative uncertainty, we used the average value of plasma excitation temperature and electron number density for the CF-LIBS analysis. To calculate the concentration of the ionic species present in the sample, the Saha-Boltzmann equation was employed consisting of the atomic (γ) and ionic (γ+1) concentrations of an element α [66]:

(13) $$N_{e}\frac{C^{\alpha ,\gamma +1}}{C^{\alpha ,\gamma }}=6.04\times 10^{21}\;\sqrt[{3}]{T_{eV}}\;\frac{Z_{\alpha ,\gamma +1}}{Z_{\alpha ,\gamma }}\;\exp\left[-\frac{\chi_{\alpha ,\gamma }}{k_{B}T}\right]$$

In Eq. (13), $C^{\alpha ,\gamma +1}$ corresponds to the concentration of the charge state γ+1, $\chi_{\alpha ,\gamma }$ is the ionization energy of the element α in (eV), $N_{e}\;\left({cm}^{-3}\right)$ is the electron number density, $Z_{\alpha ,\gamma }$ and $Z_{\alpha ,\gamma +1}$ are the partition functions of the lower charge state (γ) and upper charge state (γ+1), respectively. The elemental composition of any element in a sample is the total of the ionized ($C^{\alpha ,\gamma +1}$) and neutral ($C^{\alpha ,\gamma }$) contributions [65] and given in Eq. (14).

(14) $$C_{tot}^{\alpha ,\gamma }=C^{\alpha ,\gamma }+C^{\alpha ,\gamma +1}$$

We used the following relation to calculate the elemental percentage composition of all the elements present in the PR samples.

(15) $$C^{\alpha }\left(\mu g/g\right)=\frac{C^{\alpha }}{\sum C_{tot}^{\alpha ,\gamma }}\times 10^{6}$$

In Eq. (15), $C_{total}^{\alpha ,\gamma }$ is the relative composition of each component and $\sum C_{total}^{\alpha ,\gamma }$ is the sum of compositions of all components present in the samples.

Relative standard deviation (RSD) is the calculation of accuracy in data analysis. The RSD of statistics has close relationship with standard deviation (SD). By considering the 10 spectra of each sample, we have determined the average elemental composition of each PR sample by keeping the identical conditions as well as optimized LIBS parameters. To minimize the relative uncertainty in composition (μg/g), we measured the relative standard deviation error (RSDE) in composition (μg/g) of each component present represents the sample [13,20,67].

(16) $$\eta ={\left|\frac{\Sigma {\left(\left(x_{i}-\rho \right)\right)}^{2}}{N}\right|}^{\frac{1}{2}}$$

(17) $$RSDE\;\left(\mu g/g\right)=\frac{\eta }{\rho }\times 10^{6}$$

In Eqs. (16), (17)), ηrepresents the standard deviation, $x_{i}$ shoes the value from each data, ρ is the mean value of the data and N indicates the total number of the values.

In Table 2, we present the relative chemical concentration in (μg/g) of the detected trace REEs of the 5 PR samples determined using the CF‒LIBS analytical technique. The elemental composition (μg/g) by the CF‒LIBS method shows that elements including La, Ce, Nd, Sm, and Yb are present as minor ingredients in the PR samples. In all the PR samples, La has a comparatively higher concentration from (38.2 ± 0.012) to (52.8 ± 0.025) μg/g while Yb has a lower concentration from (1.3 ± 0.024) to (3.3 ± 0.034) μg/g. The relative standard deviation error (RSDE) of the average chemical composition (μg/g) was determined to improve the accuracy of the CF-LIBS technique as shown in Table 2. For instance, a maximum value of RSDE for all the detected REEs includes La (±0.031), Ce (±0.076), Nd (±0.029), Sm (±0.093), and Yb (±0.034). The variant in REEs chemical concentration in all PR samples might be owing to the geography of the region as well as several physical activities for the growth of phosphorite rocks that cause in reduction and enrichment of the REEs.

**Table 2:** Concentration (μg/g) of REEs in phosphorite rocks samples using CF-LIBS.

REEs	PR-(S1) (μg/g)±RSD	PR-(S2) (μg/g)±RSD	PR-(S3) (μg/g)±RSD	PR-(S4) (μg/g)±RSD	PR-(S5) (μg/g)±RSD
La	38.2 ± 0.012	42.7 ± 0.031	43.6 ± 0.011	52.8 ± 0.025	48.2 ± 0.022
Ce	31.1 ± 0.046	36.4 ± 0.061	34.1 ± 0.021	39.7 ± 0.076	29.4 ± 0.016
Nd	24.2 ± 0.029	22.3 ± 0.021	17.5 ± 0.012	25.9 ± 0.019	20.6 ± 0.025
Sm	23.7 ± 0.093	15.9 ± 0.021	17.3 ± 0.073	21.1 ± 0.033	28.8 ± 0.053
Yb	1.3 ± 0.024	2.7 ± 0.026	1.8 ± 0.021	2.4 ± 0.014	3.3 ± 0.034

In Table 3, we present a comparison between REEs content of regional phosphate rocks with internationally reported results. The comparison shows that the PR deposits of Russia represent the highest chemical concentration values for some REEs such as La (2150 μg/g), and Ce (3420 μg/g). However, Israel phosphate rocks were reported in low chemical concentrations for all REEs such as La (34 ± 1 μg/g), Ce (22 ± 4 μg/g), and Sm (5.2 ± 0.1 μg/g). Similarly, Nd is reported in lower as well as higher concentrations, for example, the Nd testified values for the USA, Egypt, Tunisia, and China are 5 ± 0.7 μg/g, 6 ± 0.6 μg/g, 120.73 (average)μg/g, and 210.67 (average)μg/g, respectively. Overall, the average calculated values in the present work for Yb, Sm, Ce, and La are closest to the statistics quoted for (USA, Israel, Syria), (Morocco, Israel, Syria), (USA, Morocco, Syria), and (Israel, Syria), respectively.

**Table 3:** Worldwide distribution of REEs in phosphorite deposits.

REEs	Present Work	USA^a^	Russia^a^	Israel^a^	Algeria^b^	Nigeria^c^	Syria^d^	Morocco^b^
Concentration (μg/g)								
La	38.2–52.8	78 ± 2	2150 ± 20	34 ± 1	191 ± 2	243 ± 0.4	46 ± 1	109 ± 2
Ce	29.4–39.7	114 ± 3	3420 ± 37	22 ± 4	290 ± 4	474 ± 2	41 ± 3	46 ± 3
Sm	15.9–28.8	14.9 ± 0.2	131 ± 2	5.2 ± 0.1	36 ± 1	56 ± 1	9.3 ± 0.1	12.9 ± 0.2
Yb	1.3–3.3	8.4 ± 0.4	10.9 ± 0.4	4.6 ± 0.2	16.5 ± 0.6	29 ± 4	5.0 ± 0.3	14.5 ± 0.6
Nd								
Present Work	USA^e^	Egypt^f^	Tunisia^g^	China^h^				
17.5–25.9	5 ± 0.7	4.14 ± 0.15	120.73 (mean)	210.67 (mean)				

a [68].

b [69].

c [70].

d [71].

e [72].

f [73].

g [74].

h [75].

### 4.5 Principal component analysis

**Fig. 8:**
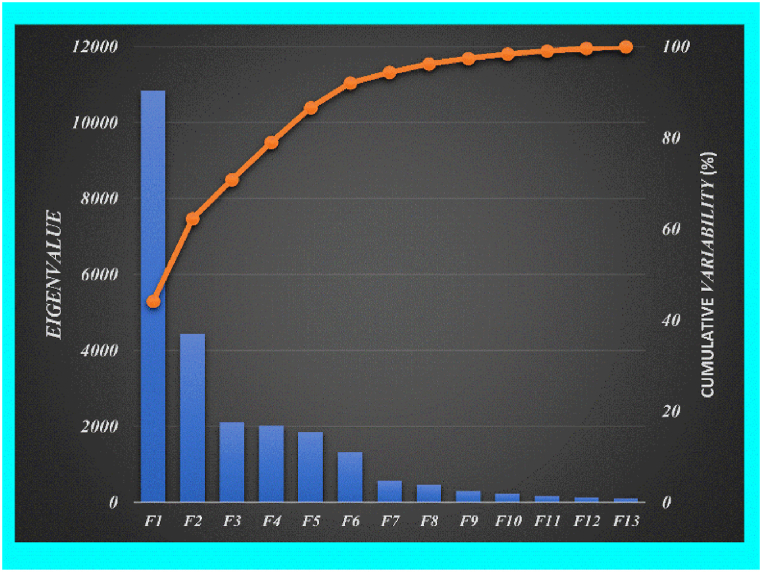
Scree plot of the eigenvalues vs each principal component factor (F) and their cumulative variability %.

To classify the various PR samples, the PCA test was utilized in this study. The data analysis software (Origin-pro/2022 (Origin Lab, USA)) was used for the PCA analysis. XLSTAT (version 2022, addinsoft, USA) was used for the pre-processing of the data employed for the PCA. PCA using LIBS spectra has been developed to recognize geological raw materials such as silicates, carbonates, and fluorites [76,77,78]. In the present work, by using the maximum spectral data covariance the PCs were constructed. In order to correct the optical emission spectra, the dark signal was subtracted from the detected signal using the LIBS software. After pre-processing of the LIBS data using background correction, one spectrum is selected as an average of 100 spectra for individual sample. Furthermore, the selected spectral data is normalized by dividing the data set by the standard deviation (SD) of each spectral data in origin-Pro2022 data analysis software. The acquired LIBS data using the Avantes Spectrometer (6-channel, CCD) is contained 24,564 spectral data points (wavelength channels) from 200 nm to 970 nm. Classification utilizing all the emission spectra of the samples can improve the estimated time as well as increase the constraints of system performance for LIBS measurements. Therefore, exclusion of highly unnecessary variables and selection of a few essential spectral emission lines of the elements is significant for multivariate analysis. All data points comprise characteristic elemental information in the target samples, however, an extremely enormous data dimension will produce complexities in computation. Therefore, it is very important to reduce properly the dimension of spectral data. PCA technique is commonly employed for analyzing and simplifying data sets such as dimension reduction of the data set, whereas data features retained that have the major variance impact. In Fig. 8, we present the interpretation rate (variance) and cumulative interpretation (cumulative variability) rate of the top 13 PCs. These PCs gives interpretation rate such as 40.70%, 22.40%, 13.20%, 5.47%, 3.87%, 3.46%, 2.41%, 1.89%, 1.59%, 1.13%, 0.97%, 0.63%, 0.14%. It can be easily observed that the total contributions of the ten PCs of the spectral data reached up to 97.86% showing the spectral variance information. The first three PCs with eigenvalues >1 have a maximum variance of the total, therefore, most of the data is explained by the first three PCs.

**Fig. 9:**
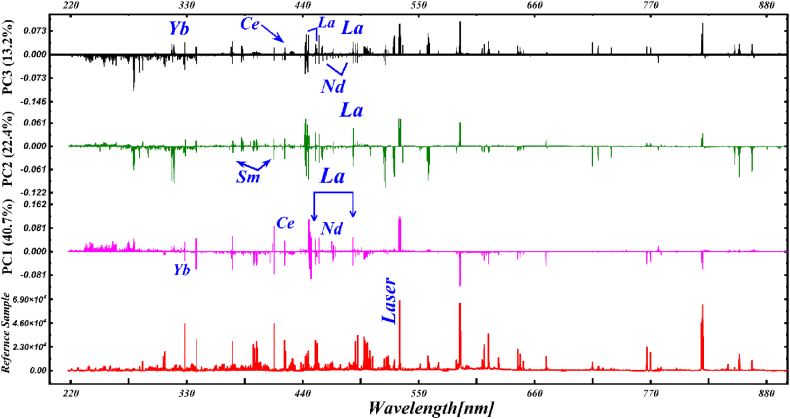
The loading plot of the first three PCs from PCA on full spectra of 5 PR samples.

**Fig. 10:**
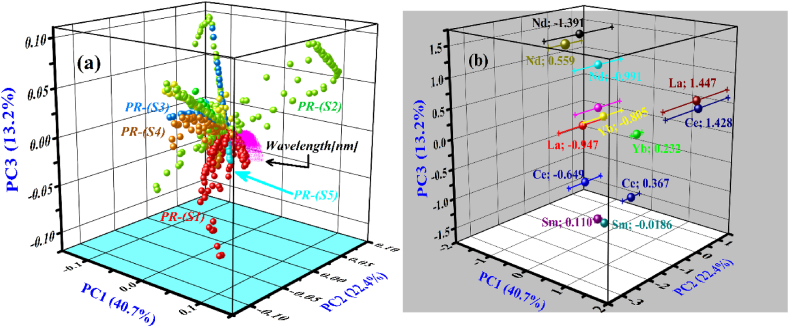
(a) 3D clustering of 5 various PR samples on the most important first three PCs, (b) 3D score plot of the first three PCs such as PC1, PC2, and PC3 for PR samples.

Initially, PCA was used to convert the full emission spectra into 13 PCs and the loading plot was accomplished to choose the important emission lines. In Fig. 9, we present the loading plots of the first three PCs showing 76.3% of the variations to the total spectral information. It can be investigated that spectral lines of trace elements including rare earths such as La, Ce, Nd, Yb, and Sm are in reasonable alignment indicating large-loading-coefficients. Furthermore, those spectral lines which they have a relatively high signal-to-noise ratio (SNR) were selected for the next statistical analysis. The selected characteristic spectral lines of the rare earths such as La II 489.991 nm, Ce II 422.260 nm, Nd I 468.345 nm, Sm II 392.240 nm, and Yb II 328.937 nm along with rich lines of Si, Ca & Ti were used to construct the matrix of the order of 140×5 (LIBS spectra × # of selected lines). In the next phase, PCA was applied using LIBS spectra at the chosen characteristic spectral-lines to present any change between the 5 varieties of the rare earth PR samples. The initial 3 PCs described 76.3% (PC1: 40.70% and PC2: 22.40%, PC3: 13.20%) of the variations between total LIBS spectral information, and their distribution score and loading charts are displayed in Fig. 10(a and b).

For the classification of the 5 PR samples, we present a 3D score scatter chart with PC1 (X-axis), PC2 (Y-axis), and PC3 (Z-axis) as shown in Fig. 10(a). The first three PCs again showed 76.3% variation of the total spectral information with PC1: 40.70%, PC2: 22.40%, and PC3: 13.20%. Fig. 10(a) demonstrates an apparent clustering of the spectral data of the 5 various PR samples containing rare earths using PC1, PC2, and PC3. The 3D clustering plot of 5 various types of PR samples is constructed using the specific high intensity several optical emission lines. Each data point in the plot represents a set of spectral lines corresponding to the sample. Each type of PR sample was marked with separate colors for a good quality visualization. We can see a significant difference between the PR samples. However, the LIBS spectral lines from the different types could not be completely bunched simultaneously. In Fig. 10(b), the PCA scores plots using PC1, PC2, and PC3 are displayed for the various rare earth PR samples using the same experimental conditions such as 100 mJ laser energy, a 2 μs time delay between the triggering of the laser-pulse and the opening of the spectral detection window, and 10 μs integration interval. The diameter of the focused laser beam at the sample surface was ∼0.05 cm which corresponds to laser fluence of about 51 Jcm^−2^. The three-dimensional array score plot is constructed using the rich spectral lines of the La, Ce, Nd, Sm, and Yb from PR samples. We select the spectral line profiles of the REEs for the score plot because these elements have fine and high SNR of the line profiles.

### 4.6 Quantitative analysis using EDX

**Fig. 11:**
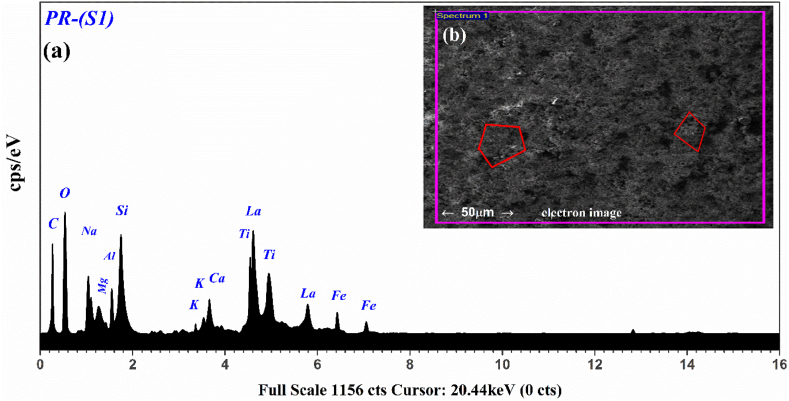
EDX spectrum of the PR sample (S1) along with microphotograph of the target sample as an inset (b).

**Fig. 12:**
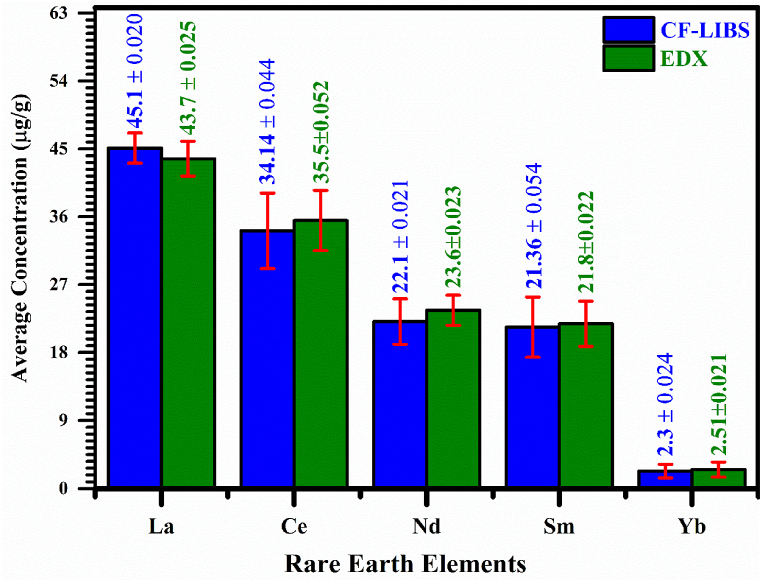
Comparison of the average chemical concentration (μg/g) obtained using CF-LIBS and EDX.

In the present study, an Oxford Instruments X-MAXN-20 EDX attached with a scanning electron microscope (SEM) having a depth-profile (1–2 μm) working at 20 keV is also used to analyze the composition of the mineral samples [28,29]. The X‒rays emitted from the target-sample were identified using a 30 mm^2^ Si (Li) detector. SEM‒EDX is a brisk technique for exploring REE-bearing minerals and distribution in complex matrix, and can be further appropriate for geological samples with a low concentration of REEs. The SEM‒EDX analysis has already been used in several fields such as biomedical, agrochemicals, metal accumulation in plants, the study of environmental pollution, classification of minerals, the study of drug nanoparticles, and metal detection in waste foundry sands. Moreover, the physical distribution of REEs is also incredibly valuable for extraction purposes. In geological samples, REE-bearing phases with other major components may appear such as correlation with raw material and contamination by soils or other mineral ingredients. Thus, both the physical distribution of REEs and chemical composition are important for the REE extractability from the geological samples [79,80,81]. We have studied all 5 PR samples using EDX analysis however, only one spectrum along with a micro-electron image of the surface scanning area up to 50 μm corresponds to the PR-(S1) is shown in Fig. 11. This figure shows the characteristic X-ray emission spectrum of the PR sample (S1) due to the K-shell and L-shell transitions of the electron. The elements such as C, O, Al, Si, Na, Mg, K, Ca, and one light rare earth element La in this EDX spectrum. Similarly, all other REEs such as Ce, Nd, Sm, and Yb in the PR samples are successfully detected by the EDX as identified in the LIBS analyses. Therefore, the multiple REEs detected by LIBS such as La, Ce, Nd, Sm, and Yb in various PR samples are validated by the EDX analysis. For comparative study, the average mass fraction (μg/g) obtained using EDX and LIBS analysis of the PR samples (S1, S2, S3, S4, and S5) is presented in Fig. 12. The error-bars (red-color) show the RSDE in the average chemical concentration (μg/g) of the 5 PR samples obtained using CF-LIBS and EDX technique. In addition, PCA, has also classified the PR samples comprising REE traces. Hence, CF-LIBS assisted with EDX, and PCA is an efficient tool to diagnose the REE-bearing phosphorite rocks.

### 4.7 LIBS and EDX detection limits

The LIBS spectroscopical analysis of materials requires a microgram sample and it minimally scratched the surface of the sample. The LIBS technique has a detection limit of about 10–50 ppm with an estimated depth profile of about 10^2^ μm. In this work, the LIBS qualitative study presented an excellent detection limit for light as well as heavy elements such as hydrogen to ytterbium (^3^H to ^70^Yb). Interestingly, LIBS results are in good agreement with that of the EDX for rare earth elements such as La, Ce, Nd, Sm, and Yb. The EDX analysis of material normally requires a conducting sample surface having a depth profile of ∼5 μm and its detection limit is ∼100 ppm for trace elements and 1000 ppm for bulk materials. Therefore, only those elements which appear in concentrations >100 ppm can be identified in the EDX analysis.

## 5 Conclusion

In the present work, the compositional analysis of the different PR samples comprising REEs as traces (μg/g) was conducted using EDX and CF-LIBS. For the qualitative analysis, atomic as well as ionic emission lines of the various ingredient elements including rare earths such as Ce, La, Nd, Sm, and Yb, have been identified in laser-produced optically-thin-plasma spectrum of PR samples using the NIST database. For the quantitative analysis, we used the CF-LIBS technique and results were compared to that obtained using EDX showing excellent agreement within 10% RSDE. Furthermore, a statistical-analysis including PCA was carried out to classify the 5 PR samples. PCA was applied using the average LIBS-spectra of PR samples consist of Ce, Nd, La, Sm, and Yb emission lines. The first three PCs were observed using LIBS-spectral data set showing a covariance up to 76.3%. It is demonstrated that LIBS assisted with EDX and PCA is a robust and reliable tool to classify and quantify the REEs in geological and mineralogical samples.

## Declarations

### Author contribution statement

Haroon Asghar: Conceived and designed the experiments; Performed the experiments; Wrote the paper.

Amir Fayyaz: Conceived and designed the experiments; Performed the experiments; Analyzed and interpreted the data; Contributed reagents, materials, analysis tools or data; Wrote the paper.

Tahani A. Alrebdi: Analyzed and interpreted the data.

A. M. Alshehri: Contributed reagents, materials, analysis tools or data.

### Funding statement

This work was supported by Princess Nourah bint Abdulrahman University Researchers Supporting Project number [PNURSP2023R71], Princess Nourah bint Abdulrahman University, Riyadh, Saudi Arabia.

This work was supported by the Deanship of Scientific Research at King Khalid University [RGP.1/340/43].

### Data availability statement

Data will be made available on request.

### Declaration of interest’s statement

The authors declare no conflict of interest.
